# A Comparative Prospective Assessment of Preoperative 5% Dextrose and Normal Saline Loading on Outcomes Following Laparoscopic Cholecystectomy

**DOI:** 10.7759/cureus.94614

**Published:** 2025-10-15

**Authors:** Tashaba Qaiser Faizi, Salman Jafferi, Anum Usman, Sadia Lateef, Rakshanda Najam Siddiqi, Adeela Zuhair Siddiqui

**Affiliations:** 1 General Surgery, Jinnah Postgraduate Medical Centre, Karachi, PAK; 2 General Surgery, University Hospitals Birmingham NHS Foundation Trust, Birmingham, GBR; 3 General surgery, Royal Berkshire NHS foundation Trust, Reading, GBR; 4 Acute Surgical Unit, Royal North Shore Hospital, Sydney, AUS; 5 General Surgery, Al Ahli Hospital, Doha, QAT

**Keywords:** dextrose solution, fluid therapy, laparoscopic cholecystectomy, postoperative complications, sodium chloride

## Abstract

Background: Laparoscopic cholecystectomy is still accompanied by postoperative pain, nausea, and vomiting, and the use of intravenous dextrose, though suggested to decrease the mentioned complications, has not been confirmed yet. The purpose of the study was to evaluate the effects of intravenous preoperative 5% dextrose and normal saline on postoperative pain, nausea, vomiting, and discharge fitness in patients undergoing elective laparoscopic cholecystectomy.

Methods: 100 patients undergoing laparoscopic cholecystectomy were involved in a comparative prospective study. The subjects were divided into two categories, i.e., Group A (dextrose, n=50) and Group B (normal saline, n=50). Demographic and clinical factors, such as age, gender, American Society of Anesthesiologists (ASA) status, and BMI, were taken. Demographic factors were further stratified into groups of postoperative outcomes of abdominal pain, nausea, vomiting, and fitness at discharge. The p-value was set at p<0.05.

Results: The average age was 44.3 ± 9 years, and the females constituted 75 % of the participants in the study. There were no differences in age, sex, ASA status, or BMI (p>0.05). The abdominal pain during the postoperative period (20% vs. 52% p=0.001) and nausea (14% vs. 36% p=0.011) were significantly increased in the saline group than the dextrose group. There was no statistically significant difference in vomiting (20% vs. 30%, p=0.248) or discharge fitness (58% vs. 48%, p=0.316). Subgroup analyses revealed significant correlations of both pain and nausea with female sex, status of ASA I, and BMI.

Conclusion: Dextrose intravenous preoperative loading was found to be better than normal saline in reducing postoperative abdominal pain and nausea, which indicated that intravenous dextrose preoperative loading was an inexpensive approach to relieve postoperative pain and nausea after laparoscopic cholecystectomy.

## Introduction

Gallstone disease is among the most common gastrointestinal diseases in the world, and laparoscopic cholecystectomy has been the best intervention for symptomatic cholelithiasis since the procedure is less invasive, has a short duration of recovery, and less morbidity as compared to open procedures [1,2]. Although a patient is unlikely to experience any adverse effects due to such a procedure, postoperative complications include abdominal pain, nausea, vomiting, and hemodynamic instability, which can seriously affect the recovery and extend the hospital stay [3]. Thus, perioperative and fluid management have been the center of clinical research studies to enhance the outcome of surgery.

IVFs are important in preserving the intravascular volumetric status, perfusion of the tissues, and reducing electrolyte imbalances during anesthesia and surgery [4]. The most frequently used fluids are normal saline (0.9% sodium chloride), but its chloride concentration is too high and could lead to hyperchloremic acidosis and slow recovery in patients [5]. By contrast, dextrose-containing solutions offer hydration and metabolic substrate, which may decrease the catabolic stress, stabilize hemodynamics, and enhance the recovery of the postoperative period [6]. Earlier research has mostly evaluated postoperative pain, nausea, and vomiting as they relate to fluid administration, but the results are inconclusive [7,8]. However, the comparison of dextrose and normal saline preload before laparoscopic cholecystectomy is limited and irregular, especially in the pain management, hemodynamic stability, and metabolism findings [9].

The current study was intended as a prospective comparative study to compare the effect of intravenous loading of 5% dextrose saline preoperatively with the effect of intravenous loading of 0.9% normal saline on the outcomes of laparoscopic cholecystectomy patients. The outcomes were postoperative nausea or vomiting, abdominal pain, and discharge fitness.

## Materials and methods

This comparative prospective study was carried out at the Department of General Surgery of Jinnah Postgraduate Medical Center Karachi between September 2021 and February 2022 (Ref: 46894/JPMC) through a consecutive sampling method after informed consent. All the patients were scheduled to undergo elective laparoscopic cholecystectomy. OpenEpi version 3.0.0 (Atlanta, GA, USA) was used to calculate the sample size of 100 based on the 95% confidence, 5% margin of error, and 30% difference in incidence of postoperative nausea and vomiting between the two groups expected [10].

Inclusion criteria were adult patients aged 18-60 years, males and females, American Society of Anesthesiologists (ASA) physical status I and II, and elective laparoscopic cholecystectomy under general anesthesia [11]. These were selected criteria to arrive at a relatively homogeneous sample with low baseline comorbidity, thus minimizing confounding variables that would independently affect the outcome of the perioperative process, including nausea or pain. The exclusion criteria were uncontrolled diabetes mellitus (leading to the altered glucose metabolism), renal or hepatic failures (that may affect the drug metabolism and fluid treatment), ischemic heart disease (to reduce the risk of perioperative heart failure), and electrolyte imbalance (that may be aggravated by fluid intake). Pregnant and lactating women were eliminated because of altered physiology and a moral concern. Patients who had undergone antiemetic or steroid treatment within 48 hours before the surgery were not included due to the risk of confounding the effects of postoperative nausea and vomiting.

Therefore, eligible subjects were subsequently randomly assigned to two groups according to the preoperative fluid administered. Group A (n = 50) was subjected to 500 mL of 5% dextrose solution injected intravenously, and Group B (n = 50) was exposed to 500 mL of 0.9% normal saline injected intravenously. Infusion of the two fluids was done 30 minutes prior to anesthesia induction. Outcome measures included the incidence of postoperative nausea, vomiting, abdominal pain intensity, and clinical readiness for discharge based on standardized institutional criteria.

Postoperative pain was evaluated based on patient-reported port-site discomfort within the first 12 hours after surgery, using standard nursing pain documentation charts. To reduce the variability of aesthetic and surgical protocols, the induction agents, maintenance anesthesia, perioperative monitoring, and postoperative analgesia were standardized. Dependent variables included postoperative nausea incidence, vomiting, severity, and clinical discharge readiness of abdominal pains based on standard hospital requirements.

Analysis of data was done using SPSS 26.0 (IBM Corp., NY, 2019). The independent t-tests were implemented on the continuous variables, while the chi-square tests were implemented on the categorical variables between the groups. The p-value of less than 0.05 was regarded as statistically significant.

## Results

One hundred patients (50 males and 50 females) undergoing elective laparoscopic cholecystectomy were recruited into the study, and they were divided into two groups (A and B). The two groups were similar in terms of their characteristics at baseline, as presented in Table 1. The average age of the participants was 44.29 ± 8.96 years, and there was no significant difference between groups (p = 0.618). Sixty-one (61%) of the patients were aged 41-60 years. The sample population was dominated by 75 (75%) of the females, with no significant differences between the groups with regard to gender (p = 0.817). Status distribution was also equal, with ASA I constituting 87 (87%) patients. The average BMI was 24.83± 2.20 kg/m^2^, and 33 (33 %) of the patients were found to be obese (>30 kg/m^2^).

**Table 1:** Baseline Characteristics of Study Participants (n = 100) Group A: Dextrose infusion; Group B: Normal saline infusion. ASA: American Society of Anesthesiologists. df: Degree of Freedom 1

Parameter	Group A (n = 50) N (%)	Group B (n = 50) N (%)	Total (n = 100) N (%)	Statistical Test	Test Value	p-value
Age (years) (Mean ± SD)	43.84 ± 9.38	44.74 ± 8.60	44.29 ± 8.96	t-test	0.50	0.618
Age Groups						
20–40 years	21 (42%)	18 (36%)	39 (39%)	Chi-square	0.38^df^	0.539
41–60 years	29 (58%)	32 (64%)	61 (61%)			
Gender						
Male	12 (24%)	13 (26%)	25 (25%)	Chi-square	0.05^df^	0.817
Female	38 (76%)	37 (74%)	75 (75%)			
American Society of Anesthesiologists Status						
I	43 (86%)	44 (88%)	87 (87%)	Chi-square	0.09^df^	0.766
II	7 (14%)	6 (12%)	13 (13%)			
Body Mass Index Status						
BMI (kg/m²) (Mean ± SD)	25.15 ± 2.23	24.52 ± 2.15	24.83 ± 2.20	t-test	1.42	0.159
Body Mass Index Categories Status						
< 30	36 (72%)	31 (62%)	67 (67%)	Chi-square	1.13^df^	0.288
≥ 30	14 (28%)	19 (38%)	33 (33%)			

There was no significant difference between groups in baseline parameters (p > 0.05), so the outcome comparisons are homogeneous. Table 2 summarizes postoperative outcomes. An occurrence of abdominal pain was also more prominent in Group B (52%) than in Group A (20%, p = 0.001). Fitness for discharge already exceeded in Group A, having 29 (58%) patients, than that of Group B with 24 (48%) patients, but the difference did not reach statistical significance (p = 0.316).

**Table 2:** Comparison of postoperative outcomes between groups (n = 100) Data is presented as mean ± SD or frequency (percentage). Group A: Dextrose infusion; Group B: Normal saline infusion *Significant at p < 0.05.

Postoperative Outcome	Group A (n = 50) N (%)	Group B (n = 50) N (%)	Total (n = 100) N (%)	Statistical Test	Test Value	p-value
Abdominal pain	10 (20%)	26 (52%)	36 (36%)	Chi-square	10.37	0.001*
Nausea	7 (14%)	18 (36%)	25 (25%)	Chi-square	6.47	0.011*
Vomiting	10 (20%)	15 (30%)	25 (25%)	Chi-square	1.34	0.248
Fitness at discharge	29 (58%)	24 (48%)	53 (53%)	Chi-square	1.01	0.316

Table 3 describes the subgroup analyses. Abdominal pain was significantly related to age ≥41 years (p = 0.008), female gender (p = 0.002), ASA I and II (p = 0.023 and p = 0.005), and BMI < 30 kg/m^2^ (p = 0.001). Female gender (p = 0.026) and ASA I status (p = 0.014) exhibited a significant relation to nausea, and vomiting exhibited a significant association with ASA II status (p = 0.021). There was no significant impact of age, gender, ASA status, or BMI on fitness to discharge from the hospital (p > 0.05).

**Table 3:** Comparison between groups with respect to selected variables (n = 100) Data is presented as frequency (percentage) or mean ± SD. Group A: Dextrose infusion; Group B: Normal saline infusion, Chi-Square test: data set ≥5, Fisher’s exact test: data set <5. *Significant at p < 0.05.

Variable	Group A, N (%)	Group B, N (%)	Total N (%)	Statistical Test	Test Value	p-value
Abdominal Pain						
Age 20–40 yrs	4 (19%)	8 (44.4%)	12 (30.8%)	Fisher’s exact	1.50	0.700
Age 41–60 yrs	6 (20.7%)	18 (56.3%)	24 (39.3%)	Chi-square	7.05	0.008*
Male	2 (16.7%)	5 (38.5%)	7 (28%)	Fisher’s exact	1.05	1.000
Female	8 (21.1%)	21 (56.8%)	29 (38.7%)	Chi-square	9.47	0.002*
ASA I	9 (20.9%)	20 (45.5%)	29 (33.3%)	Chi-square	5.16	0.023*
ASA II	1 (14.3%)	6 (100%)	7 (53.8%)	Fisher’s exact	2.70	0.005*
BMI <30	5 (13.9%)	18 (58.1%)	23 (34.3%)	Chi-square	11.8	0.001*
BMI ≥30	5 (35.7%)	8 (42.1%)	13 (39.4%)	Fisher’s exact	0.44	0.999
Nausea						
Age 20–40 yrs	3 (14.3%)	7 (38.9%)	10 (25.6%)	Fisher’s exact	1.18	0.083
Age 41–60 yrs	4 (13.8%)	11 (34.4%)	15 (24.6%)	Chi-square	3.63	0.057
Male	3 (25%)	6 (46.2%)	9 (36%)	Fisher’s exact	1.50	0.411
Female	4 (10.4%)	12 (32.4%)	16 (21.3%)	Chi-square	5.01	0.026*
ASA I	6 (14%)	17 (38.6%)	23 (26.4%)	Chi-square	6.04	0.014*
ASA II	1 (14.3%)	1 (16.7%)	2 (15.4%)	Fisher’s exact	0.35	0.999
BMI <30	6 (16.7%)	12 (38.7%)	18 (26.9%)	Chi-square	3.54	0.060
BMI ≥30	1 (7.1%)	6 (31.6%)	7 (21.2%)	Fisher’s exact	3.00	0.195
Vomiting						
Age 20–40 yrs	5 (23.8%)	3 (16.7%)	8 (20.5%)	Fisher’s exact	4.00	0.702
Age 41–60 yrs	5 (17.2%)	12 (37.5%)	17 (27.9%)	Chi-square	2.80	0.094
Male	4 (33.3%)	4 (30.8%)	8 (32%)	Fisher’s exact	1.83	0.999
Female	6 (15.8%)	11 (29.7%)	17 (22.7%)	Chi-square	1.82	0.176
ASA I	10 (23.3%)	11 (25%)	21 (24.1%)	Fisher’s exact	0.61	0.999
ASA II	0 (0%)	4 (66.7%)	4 (30.8%)	Fisher’s exact	∞	0.021*
BMI <30	9 (25%)	11 (35.5%)	20 (29.9%)	Chi-square	0.63	0.426
BMI ≥30	1 (7.1%)	4 (21.1%)	5 (15.2%)	Fisher’s exact	3.27	0.366
Fitness at Discharge						
Age 20–40 yrs	12 (57.1%)	9 (50%)	21 (53.8%)	Chi-square	0.08	0.752
Age 41–60 yrs	17 (58.6%)	15 (46.9%)	32 (52.5%)	Chi-square	0.58	0.444
Male	4 (33.3%)	5 (38.5%)	9 (36%)	Fisher’s exact	0.61	0.999
Female	25 (65.8%)	19 (51.4%)	44 (58.7%)	Chi-square	1.35	0.245
ASA I	25 (58.1%)	23 (52.3%)	48 (55.2%)	Chi-square	0.18	0.668
ASA II	4 (57.1%)	1 (16.7%)	5 (38.5%)	Fisher’s exact	0.27	0.266
BMI <30	19 (52.8%)	15 (48.4%)	34 (50.7%)	Chi-square	0.06	0.808
BMI ≥30	10 (71.4%)	9 (47.4%)	19 (57.6%)	Chi-square	1.14	0.286

In general, postoperative abdominal pain and nausea levels were much higher in Group B than in Group A, and subgroup analyses revealed that female gender, increased ASA status, and reduced BMI could be considered the predictors of poor outcomes, but there was no difference in terms of fitness to leave the hospital.

## Discussion

This was a prospective study that compared the impact of intravenous loading with 5% dextrose or normal saline when administered preoperatively in patients with laparoscopic cholecystectomy on the postoperative outcomes. It was found that patients in the dextrose group had significantly less postoperative abdominal pain and nausea. The comparison between groups was valid since demographic variables such as age, gender, ASA status, and BMI were similar. The findings indicate that preoperative dextrose loading could be used to promote the comfort of patients during the early postoperative phase.

Several factors that contribute to pain in laparoscopic cholecystectomy have been identified as residual pneumoperitoneum, diaphragmatic irritation, and inflammatory reactions [12]. Stabilization of the cellular metabolism and the sensitization of nociceptors to stressor effects have been suggested by adequate hydration and glucose supplement [13]. The outcome of lower abdominal pains in the dextrose group of our research is in agreement with other research works, which emphasize the effect of carbohydrate-saturated solutions in reducing catabolic stress and enhancing postoperative recovery [14]. Dextrose can facilitate the attainment of pain reduction since it can alleviate the early postoperative catabolism and tissue hypoperfusion through the provision of an exogenous source of glucose [15].

The most unpleasant complications after laparoscopic surgeries include nausea and vomiting. Nausea in this study was much lower with the dextrose group, although there was no difference between vomiting in the groups [16]. This result is consistent with prior evidence that preoperative administration of dextrose may reduce nausea by balancing perioperative glucose levels to prevent hypoglycemia, a known cause of nausea [17]. This lack of significant difference in vomiting could be attributed to multiple etiological factors of the condition, such as anesthetic medication, intra-abdominal pressure, and predisposition of patients. Thus, dextrose loading seems to protect against nausea, but its effect on vomiting is unclear [18].

There were no major differences in discharge, whether dextrose reduced discomfort or not, but that does not mean that it does not minimize the time needed to safely discharge [19]. It has also been noted in previous literature that perioperative carbohydrate loading enhances patient satisfaction and decreases thirst or hunger but does not always affect discharge readiness [20]. The positive outcomes of preoperative dextrose loading can be attributed to a number of physiological processes. Dextrose is an immediate source of energy that assists in the maintenance of plasma glucose levels and lowers the catabolic stress response, which consequently could diminish the discharge of stress hormones like cortisol and catecholamines that make nociceptors hypersensitive. Moreover, maintenance of stable glucose levels averts nausea caused by perioperative hypoglycemia and enhances hemodynamic stability, thus leading to alleviation of postoperative pain.

Despite the fact that our study has shown a positive result of dextrose loading on postoperative outcomes, the relatively small sample size might have reduced statistical power for less frequent outcomes. Moreover, no objective measurement of hydration status or fasting duration was made, and this may have had an impact on the postoperative symptoms of pain and nausea. The limitations of this study are that it is a single-center study, so it might not be very generalizable. Second, biochemical parameters like perioperative glucose, insulin resistance indexes, and electrolyte shifts were not assessed, which could have been meaningful in terms of mechanism. Third, the follow-up was brief and did not address long-term results and complications. The next step to be taken is to incorporate dextrose loading into an enhanced recovery after surgery (ERAS) program to assess whether it can positively impact the overall quality of recovery of patients.

## Conclusions

This comparative prospective study revealed that intravenous loading of dextrose 5% preoperatively reduced the abdominal pain and nausea significantly in patients who underwent laparoscopic cholecystectomy as compared to those who were intravenously loaded with normal saline. Nonetheless, there were no noteworthy variations in postoperative vomiting as well as discharge fitness. These results indicate that dextrose infusion can be used as an inexpensive, safe, and easy-to-implement intervention to facilitate early postoperative pain.

Since the sample was small, and the study was one-centered, it would be advisable to undertake larger multi-centered studies with a longer period of follow-up to support these findings and consider their incorporation into improved recovery after surgery practices.
